# Design and *In Vitro* Interference Test of Microwave Noninvasive Blood Glucose Monitoring Sensor

**DOI:** 10.1109/TMTT.2015.2472019

**Published:** 2015-10-01

**Authors:** Heungjae Choi, Jack Naylon, Steve Luzio, Jan Beutler, James Birchall, Chris Martin, Adrian Porch

**Affiliations:** School of Engineering, Cardiff University, Cardiff CF24 3AA, Wales, U.K.; School of Engineering, Cardiff University, Cardiff CF24 3AA, Wales, U.K. He is now with the Laser Controls Systems Group, ELI Beamlines, Academy of Science, Institute of Physics, Prague, Czech Republic.; College of Medicine, Swansea University, Swansea SA2 8PP, U.K.; School of Engineering, Cardiff University, Cardiff CF24 3AA, Wales, U.K.; School of Pharmacy and Pharmaceutical Sciences, Cardiff University, Cardiff CF10 3NB, Wales, U.K.; School of Pharmacy and Pharmaceutical Sciences, Cardiff University, Cardiff CF10 3NB, Wales, U.K.; School of Engineering, Cardiff University, Cardiff CF24 3AA, Wales, U.K.

**Keywords:** Biomedical sensors, dielectric measurements, emerging application for RF/microwaves, material characterization, microwave sensors

## Abstract

A design of a microwave noninvasive continuous blood glucose monitoring sensor and its interference test results are presented. The novelty of the proposed sensor is that it comprises two spatially separated split-ring resonators, where one interacts with the change in glucose level of a sample under test while the other ring is used as a reference. The reference ring has a slightly different resonant frequency and is desensitized to the sample owing to its location, thus allowing changes in temperature to be calibrated out. From an oral glucose tolerance test with two additional commercially available sensors (blood strip and continuous glucose monitor) in parallel, we obtained encouraging performance for our sensor comparable with those of the commercial sensors. The effects of endogenous interferents common to all subjects, i.e., common sugars, vitamins (ascorbic acid), and metabolites (uric acid) have also been investigated by using a large Franz cell assembly. From the interference test, it is shown that the change in sensor response is dominated by changes in glucose level for concentrations relevant to blood, and the effects of interferents are negligible in comparison.

## I. Introduction

Diabetes is a huge worldwide problem whose prevalence is rapidly increasing. The number of people with diabetes is estimated to increase to 5 million in 2025 in the U.K. only [1], and 366 million in 2030 worldwide [2]. Due to limited capabilities of public healthcare systems, it is necessary for people with diabetes to be able to self-monitor their own blood glucose levels. Blood glucose meters using blood strips with finger pricking lancets are the most common method widely available because of their reasonably high accuracy and precision, high convenience, low cost, and their ability to provide real-time plasma glucose levels on demand. However, fingers can become painful if regular testing is needed (as in people with type I diabetes) and this type of testing can be more of a problem for some patient groups, e.g., infants.

Over the last few decades, a variety of minimally or noninvasive blood glucose monitor (NIBGM) devices based on a wide range of interesting technologies have been introduced [3]–[5]. To name a few of the most recent technologies, a transdermal glucose sensor using ultrasound [6], a small implantable fluorescent technology-based sensor combined with a body-worn data transmitter [7], a watch-type sensor [8], an ultrasound-based ear-clip type noninvasive multi-sensor for discrete measurement [9], contact-lens-type noninvasive sensor [10], occlusion spectroscopy-based sensor measuring blood flow [11], and electrochemical subcutaneous continuous monitoring sensors [12] have been developed, pursuing their way towards obtaining necessary approval to enter the market. In the RF and microwave area, the near-field monitoring technique has been widely used for its noninvasive characteristic. A broadband coplanar waveguide sensor with the combination of microfluidic system [13], an open-ended spiral-shaped microstrip transmission line sensor [14], microstrip patch/slot antenna-based sensor [15]–[17], and millimeter-wave waveguide transmission measurement system [18] have been reported using microwave technique. The basis of these is that the complex dielectric constant changes with the presence and fluctuation of blood glucose concentration in the water/glucose system [19], [20].

We have chosen microwaves as the technological platform for our NIBGM sensor since resonance-based microwave sensors have been used extensively in the precise dielectric characterization of organic and inorganic materials in minimally invasive or noninvasive ways [21]–[25]. Furthermore, microwaves around 1 GHz penetrate the body by a few centimeters, and in the NIBGM application microwave power levels of only 1 mW are needed, so they are a nonionizing low-power exposure. Therefore, we believe that the microwave method is precise, safe, and fast, providing continuous readings at sub-second intervals if required.

In a microwave resonating structure, it is almost impossible to monitor the temperature within the resonator by conventional means because the presence of temperature measuring components (for example, a thermistor and its wires) would affect the resonance. The measured temperature is also the ambient temperature rather than the temperature of the resonating element itself, which, in this case, is the ring resonator. The novelty of the proposed sensor lies in the fact that it has a reference ring in parallel with the sensing ring. The reference ring is placed: 1) far enough from the human skin so it is not affected by a property change of the human body in the immediate vicinity of the sensor (in this case, its effective dielectric constant) and 2) far enough from the sensing ring so they are not mutually coupled to each other so that the reference ring is not affected by the property change. However, both rings are made of the same material (silver-coated copper wire), have the same gap distance, and share the same polytetrafluoroethylene (PTFE) pillar as a supporting structure. Therefore, when the temperature of the whole sensor changes, both rings will exhibit similar temperature dependences. Therefore, we are able to monitor the temperature of the proposed sensor by monitoring the resonant frequency and 3-dB bandwidth of the reference ring without having any kind of dedicated temperature sensor. The pre-characterized temperature dependence of the sensing ring is then used to calibrate out the temperature-dependent effects from the measurement.

The design criteria and equivalent circuit model of the proposed sensor is given in [27]. The added value of this paper is that more practical design aspects are discussed including 3-D sensor and body modeling, and the effects of endogenous interferents common to all subjects, e.g., common sugars, vitamins (ascorbic acid), and metabolites (uric acid) are investigated by using a large Franz cell assembly. In Section II, we will discuss the design of our microwave NIBGM sensor using split-ring resonators by focusing on the practical design aspects. The experimental setup and results of the interference tests are presented in detail in Section III.

## II. Sensor Design

### A. Double Split-Ring Resonators

Since its introduction, the split-ring resonator has been used as one of the main tools of material characterization [21]–[26]. Its key benefit is that it can easily be designed for the size and shape of a given sample rather than having to shape the sample to fit into the existing resonator, for example, as in a cavity resonator. Especially when we consider noninvasive continuous monitoring of the human body, any such sample shaping, of course, is difficult. In this work, we have chosen a lumped-element split-ring resonator because it has stronger leakage of its electric field (E-field) from the gap area. A relatively low operating frequency, around 1.4 GHz, is chosen to allow for greater penetration of E-field and greater surface area of interaction.

**Fig. 1:**
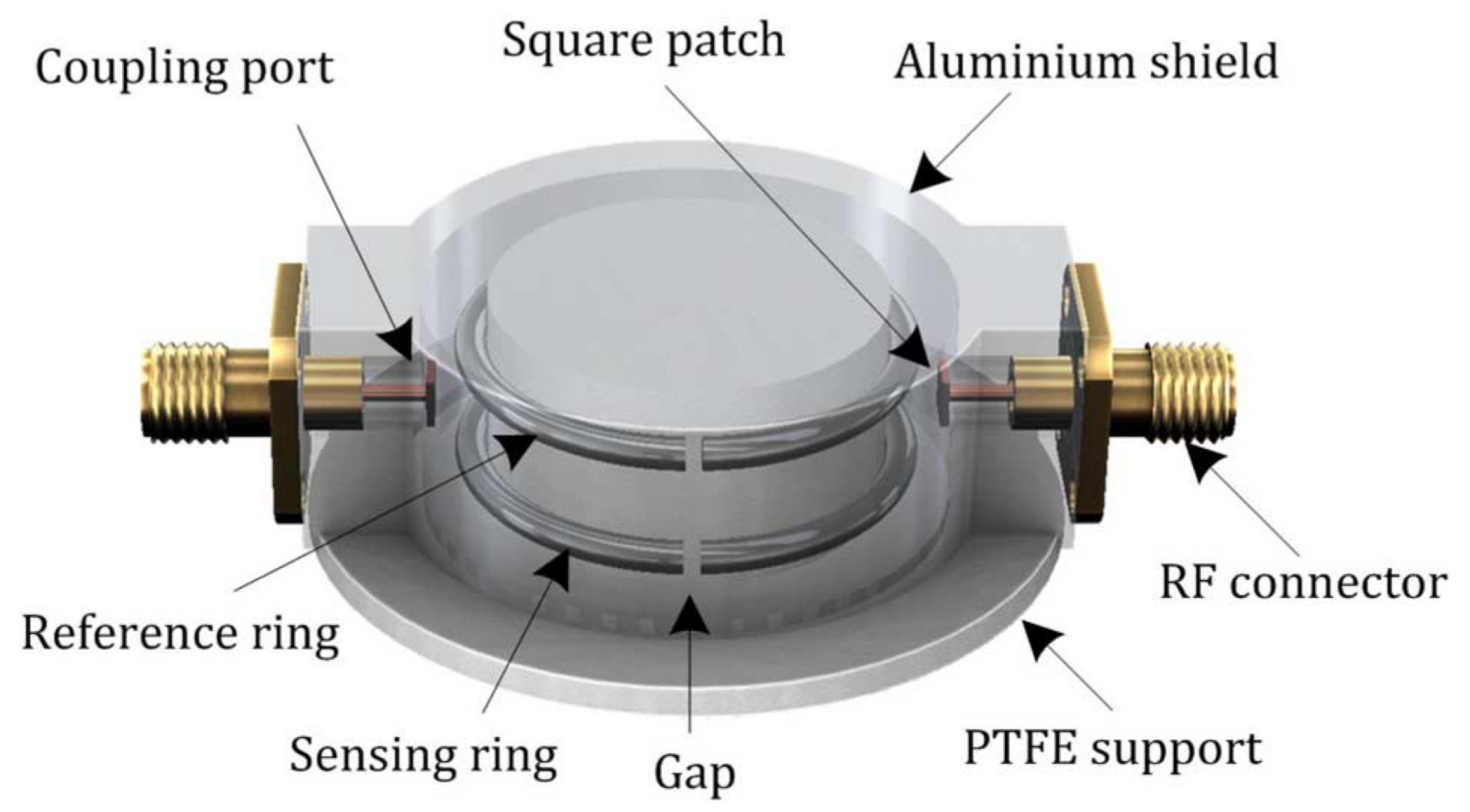
3-D structure of the proposed sensor based on discrete double split-ring resonators, after [27]. The skin side of the sensor is pointing downwards.

**Fig. 2:**
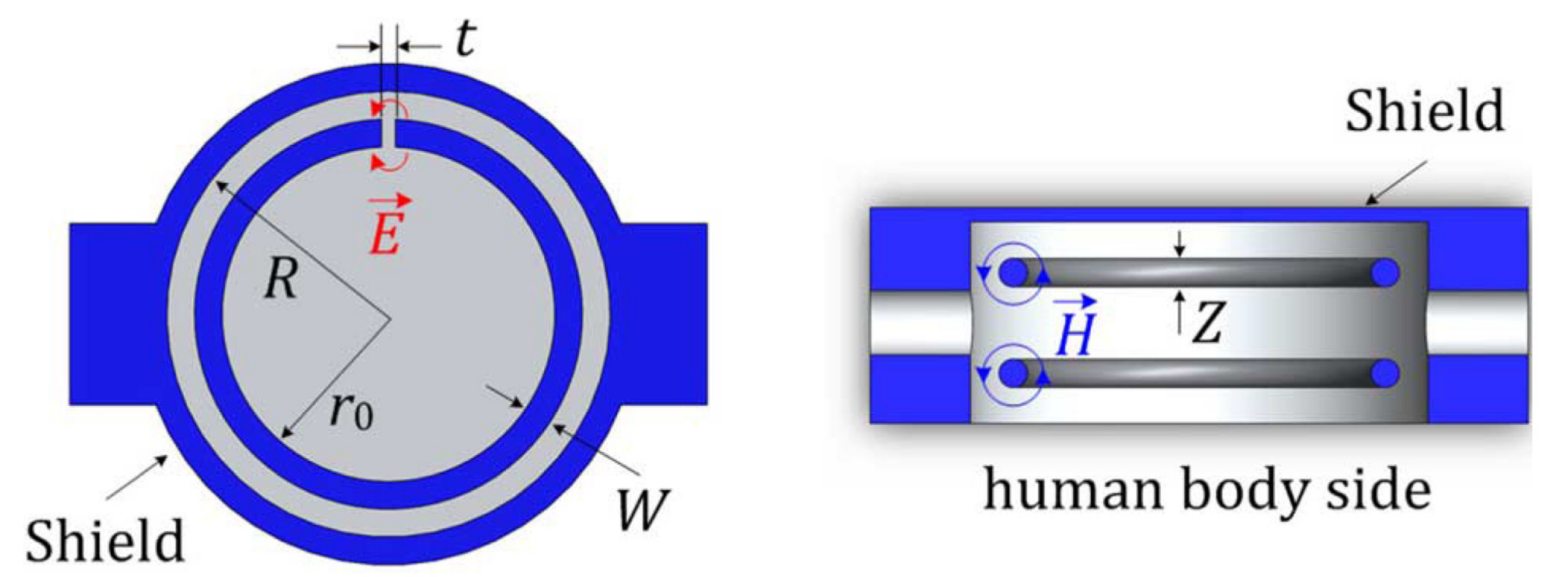
Cross-sectional top view (*left*) and side view (*right*) of the proposed sensor.

Fig. 1 shows the 3-D model of the proposed sensor based on a pair of lumped-element split-ring resonators. The ring is made from silver-coated copper wire of 1.5-mm diameter. Each ring has a gap distance of 1 mm so each forms a lumped-element *LC* resonator. The resonant frequency can be estimated from the following equation in general form [21]:

(1) $$f_{0}\approx \frac{c}{2\pi r_{0}}\sqrt{\frac{t}{\pi W}}\sqrt{1+\frac{r_{0}^{2}}{R^{2}-{\left(r_{0}+W\right)}^{2}}}\sqrt{\frac{1+\frac{\Delta Z}{Z}}{1+\frac{\Delta W}{W}}}$$

where *c* is the velocity of light, *r*_0_ is the internal radius of the ring, *W* is the gap width, *t* is the gap distance, *R* is the shield radius, *Z* is the height of the resonator, Δ*W* is the equivalent width extension of the gap due to the gap electric leakage fields, and Δ*Z* is the equivalent length extension due to the magnetic leakage fields. When the resonator is made of a single core wire, as illustrated in Fig. 2, *Z* = *W* and Δ*Z* = Δ*W*. A ring with an internal radius of 13 mm with 1-mm gap supported by a solid PTFE pillar gives a resonant frequency of approximately 1.4 GHz.

A cylindrical aluminium shield is used with one side open to confine the electromagnetic (EM) field and focus it towards the human body (on the open side). The radius of the shield is 16 mm. A hat-shaped supporting pillar made of low-loss PTFE with a radius of 13 mm is used to hold the rings in position. The EM fields of the split ring spill out of the open side of the aluminium shield, i.e., the side that will be fixed to the body; this is the bottom surface, as shown in Fig. 2.

The split-ring resonator can be coupled by both inductive and capacitive coupling techniques. To minimize the possible imbalance in the coupling strength between two ports, we have chosen the capacitive probe coupling rather than inductive loop coupling. It is also easier to fabricate a uniform probe, which is an extension of the inner conductor of the coaxial cable, than equivalent loops. To equally couple to two concentric rings that are vertically separated, a small patch is added at the end of the probe to increase the coupling strength. The antenna probe couples to the E-field in the proximity of the gap.

The two rings are wound from 1.5-mm-diameter silver-coated copper wire, with resonant frequencies offset from each other, by about 250 MHz in this case. In the current design, the resonant frequency of the sensing ring is lower than the reference ring since its frequency is reduced the most due to the dielectric loading of the body. The sensing ring is located closer to the body, while the reference ring is further away from the body. The key idea is to place the reference ring far enough from the body so that the EM field from the reference ring has minimal interaction with the body, while the EM field from the sensing ring interacts strongly. Since they have the same temperature expansion coefficient, the reference ring will provide us the change in property only due to the temperature, which can be used for temperature calibration of the measured data from the sensing ring.

**Fig. 3:**
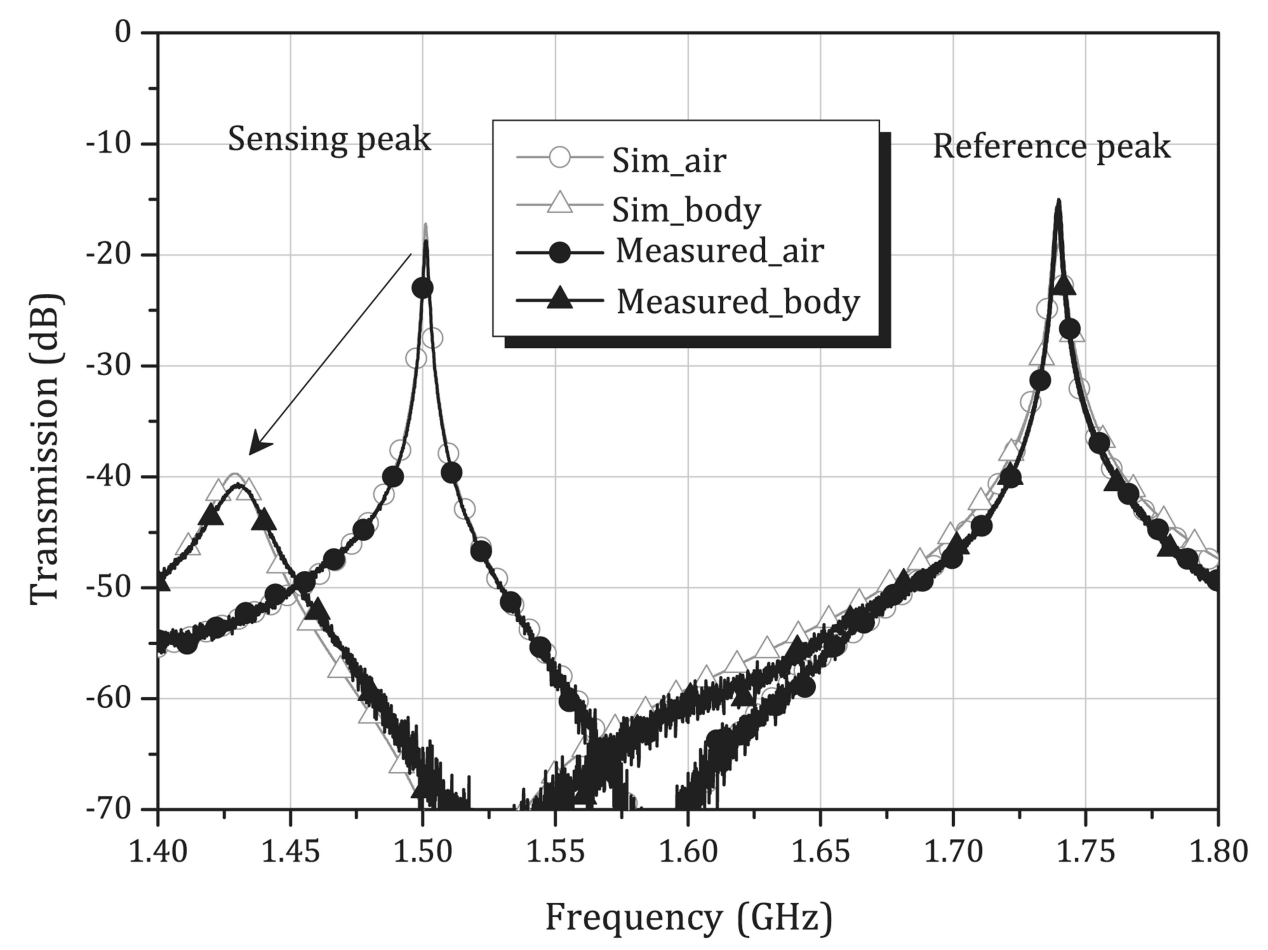
Measurement and post-simulation results of the equivalent circuit model given in [27] when the sensor is loaded with air and body. The circuit values in the simulation used to fit the measured results are as follows: *L_r_* = 6.5 *μ*H (air) and 4.185 *μ*H (body), *C_gr_* = 1.289 fF (air) and 2 fF (body), *Q_Cgr_* = 4000, *Q*_*C*gs_ = 4000 (air) and 100 (body), *L_s_* = 12 *μ*H, *C*_gs_ = 0.937 fF (air) and 1.033 fF (body), *M* = 10 nH, *C_c_* = 10 pF. Attenuators of 7.5 dB (air) and 9.5 dB (body) are added on each side of the ports to emulate coupling losses.

To visualize the sensing and reference ring operations, a simple equivalent-circuit model is presented in [27]. Measurement and simulation results are shown in Fig. 3 by using Advanced Design System (ADS) 2011 by Keysight Technology. Measurement shows the transmission characteristic when the sensor is in the air and on the body. In simulation, the change in loading condition when the sensor is on the body is emulated equivalently by varying the gap capacitance (*C*_gs_), the quality factor (*Q*_*C*gs_), and inductance (*L_s_*) of the sensing ring. The distance between the rings can be determined in such a way that: 1) the reference ring is not affected by any change in human body and 2) both rings do not mutually couple to each other, avoiding shifts in resonance frequencies and distortion of the measurement characteristics. The vertical distance between the rings is 8 mm in the proposed design. As shown in Fig. 3, the constancy of resonant peaks of the reference ring demonstrates that it is almost unaffected by the dielectric changes in the human body and also by any characteristic change in the sensing ring. The measured frequency shift of the reference peak is 600 kHz, which is a fractional frequency shift of 0.034% at 1.74 GHz. The measured *Q* of the sensing peak is about 800 when in the air, and 80 when on the body.

### B. 3-D Sensor and Body Modeling

**Fig. 4:**
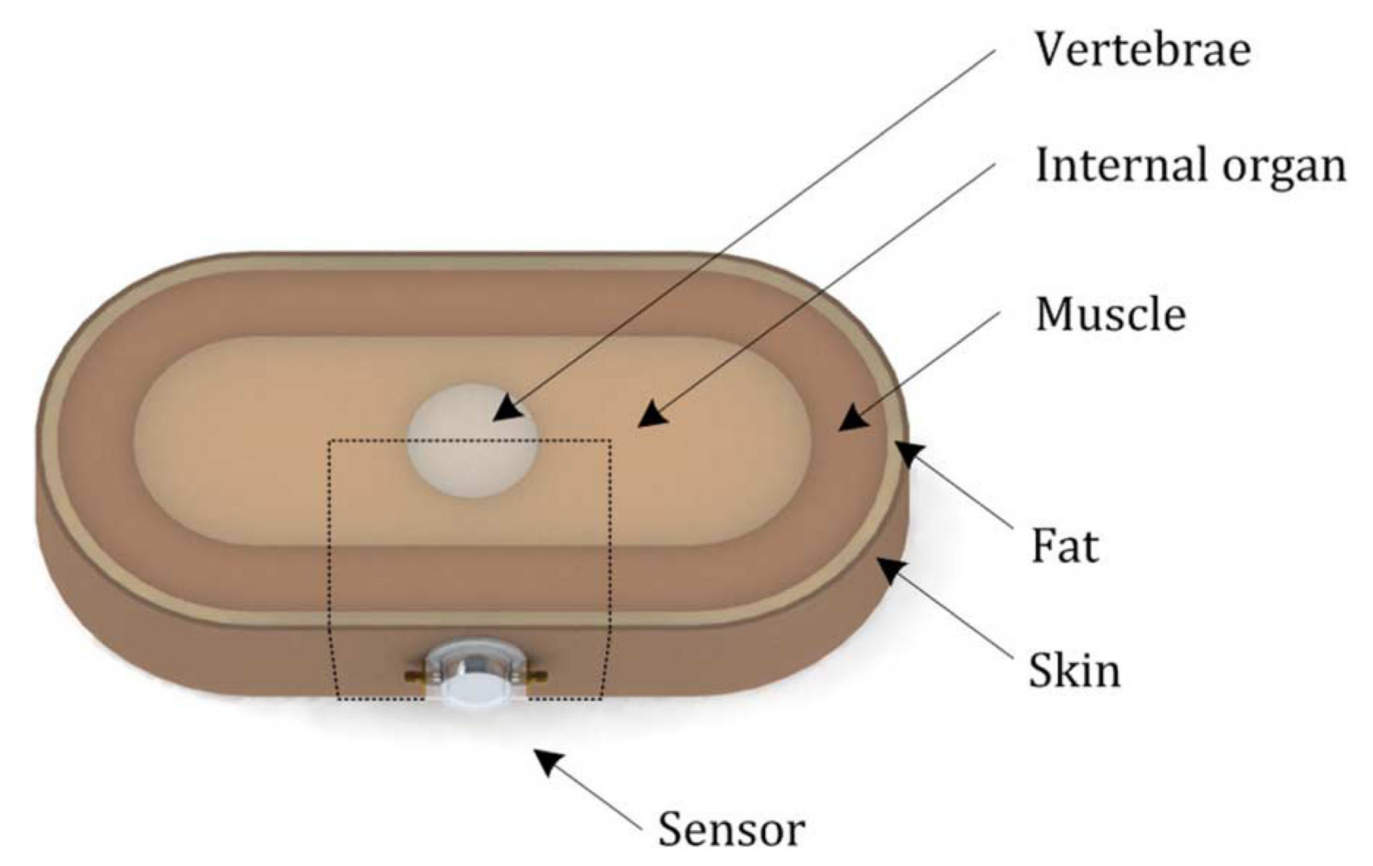
3-D abdominal cross-section model with the proposed sensor.

**Fig. 5:**
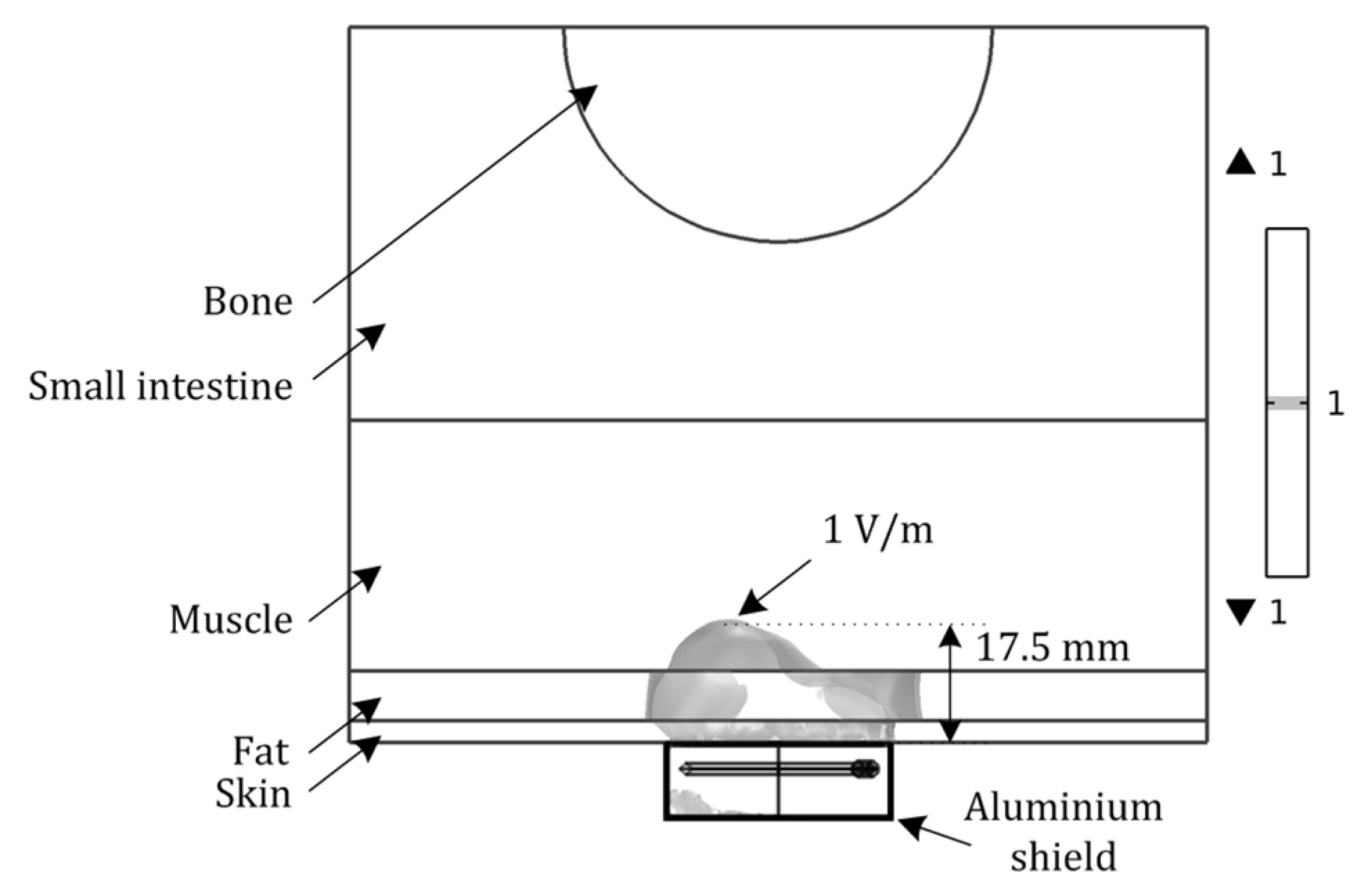
Simulated normalized E-field distribution of 1 V/m at 1.433 GHz, showing penetration depth through skin, fat, and muscle layers when excited by a microwave input power level of 1 mW.

To further investigate how the EM field produced from the ring resonator penetrates into the human body through different layers with different dielectric properties, a simple 3-D abdominal cross-section model is created in COMSOL Multiphysics v4.4, as shown in Fig. 4. The model consists of skin, fat, muscle, internal organs, and vertebrae (specifically, lumbar vertebrae) bone. The thicknesses of the skin, fat, muscle layer are 3, 7, and 35 mm, respectively, and the radius of vertebrate is 30 mm. Internal organs are assumed to fill the region between the vertebrae and muscle layer with a minimum thickness of 25 mm. The overall dimensions of the model are 400 mm × 200 mm × 60 mm, based on a healthy male adult. The dielectric properties of each layer measured at 1.4 GHz are listed in Table I, obtained from [14]. From a series of iterations, we found that only a small portion of the model where the sensor is located has direct effect on the EM-field distribution. Therefore we decided to reduce the model into the rectangular, dotted area shown in Fig. 5. After reducing the total area to 140 mm × 100 mm × 60 mm, simulation results show less than 0.5% error in resonance frequency and 0.1% error in 3-dB bandwidth, while the calculation time is greatly reduced by about 70%.

Fig. 5 shows the simulated normalized E-field intensity of 1 V/m as the iso-surface inside each layer of the proposed model. As can be expected from Table I, the E-field experiences more rapid decay at the skin and muscle layer with its high relative permittivity due to high water content, while some local expansion of the E-field is observed in the fat layer with lower loss tangent. This provides an important insight in the practical use that, even if there is a thick layer of fat, the EM field will be able to penetrate well into the skin and fat layer in the abdominal area. In this model, the E-field with an intensity of 1 V/m, for example, penetrates up to 17.5 mm from the surface of the skin when the input power is 1 mW. The penetration at each layer can be calculated by using the definition of penetration depth in case of a dielectric with very low conductivity [29],

(2) $$P_{d}=\frac{2c\sqrt{\varepsilon_{1}}}{\omega \varepsilon_{1}\;\tan\;\delta }$$

where *ε*_1_ is the real permittivity and *ε*_2_ is the imaginary permittivity of the dielectric, tan *δ* = *ε*_2_/*ε*_1_, and again, *c* is the speed of light. Table II shows the comparison of simulation and measured results of the proposed sensor when on the body, proving that the model is able to predict the measurement with reasonable accuracy.

### C. In Vivo Study—Oral Glucose Tolerance Test

The swept transmission parameter (*S*_21_) is measured in the frequency domain by using a vector network analyzer (VNA) controlled by a PC by using LabVIEW. The software finds the peak frequency and 3-dB bandwidth from the initial sweep by curve fitting a skewed Lorentzian function, then feeding them back to the instrument to reconfigure the measurement span to two times the 3-dB bandwidth for accurate tracking of property change. The skewed Lorentzian function is given by (3) [30]

(3) $$y=a_{0}+a_{1}x+\frac{a_{2}+a_{3}x}{1+a_{4}x+a_{5}x^{2}}$$

where *x* is the frequency normalized by itself to lie within [0, 1], *y* is the linear power transmission through the resonant sensor, also normalized to lie within [0, 1]. From the six fitted coefficients *a*_0_–*a*_5_, the resonant frequency is given by

(4) $$f_{0}=f_{\text{cent}}+\left(\frac{a_{1}-a_{2}}{2a_{2}}\right)\cdot f_{\text{span}}$$

and the bandwidth by

(5) $$\mathrm{BW}=f_{\text{span}}\cdot \frac{\sqrt{a_{1}^{2}-4a_{2}}}{a_{2}}.$$

For all experiments, the software configured the VNA to perform a segmented sweep with two segments of 101 points over the bandwidth of two times the 3-dB bandwidth, an IF bandwidth of 300 Hz, and source power of 0 dBm. All other settings are as per the defaults for the instrument. The two segments measured the two resonant modes of the sensor, of which only the lowest (the “sensing peak” at approximately 1.4 GHz) is used for analysis.

**Fig. 6:**
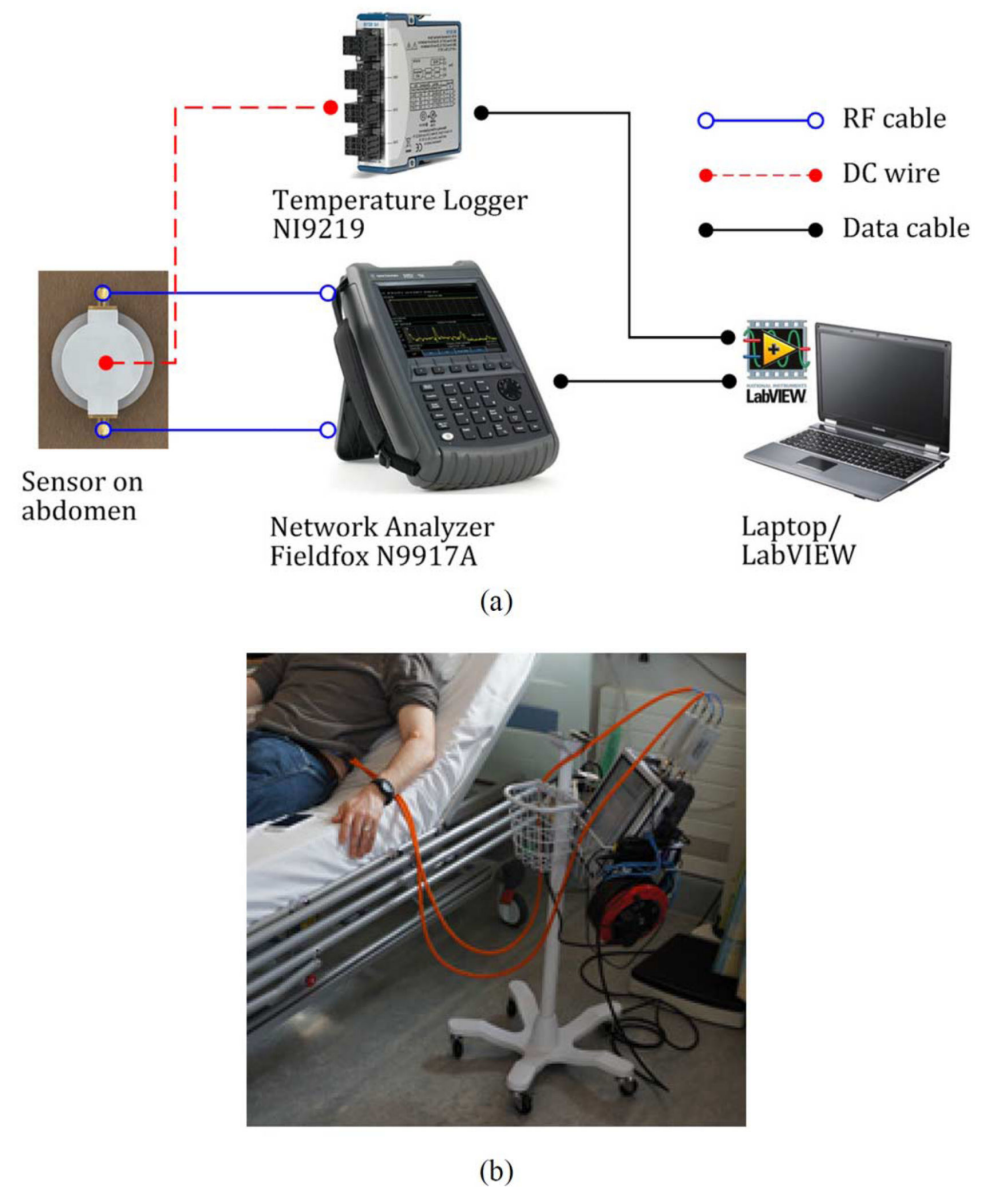
OGTT setup. (a) Schematic diagram and (b) photograph showing the system connected to the test subject.

Fig. 6(a) and (b) shows the experimental setup for an oral glucose tolerance test (OGTT). While the temperature of the aluminium shield is monitored by resistance temperature detectors (RTDs), the temperature inside the shield is monitored and calibrated by the reference ring technique explained before. For comparison, measurements of blood glucose are entered with the microwave data, obtained from a commercial blood strip device (e.g., Bayer CONTOUR), and also compared with the data produced by a continuous glucose monitoring device (e.g., Medtronic Guardian), worn by the person under test. Our sensor is attached to the abdominal skin by double-sided adhesive tape (#1510, Hi-Tack Conformable Double Coated Tape, 3M). To prevent thermal lag between the rings owing to the low thermal conductivity of the PTFE support, prior to fixing the sensor on the body we warm it to approximately 37 °C by placing it on a warm plate for around 15 min. After fixing to the body, the sensor is left for a further 30 min before readings are taken to allow it to come into thermal equilibrium with the body.

**Fig. 7:**
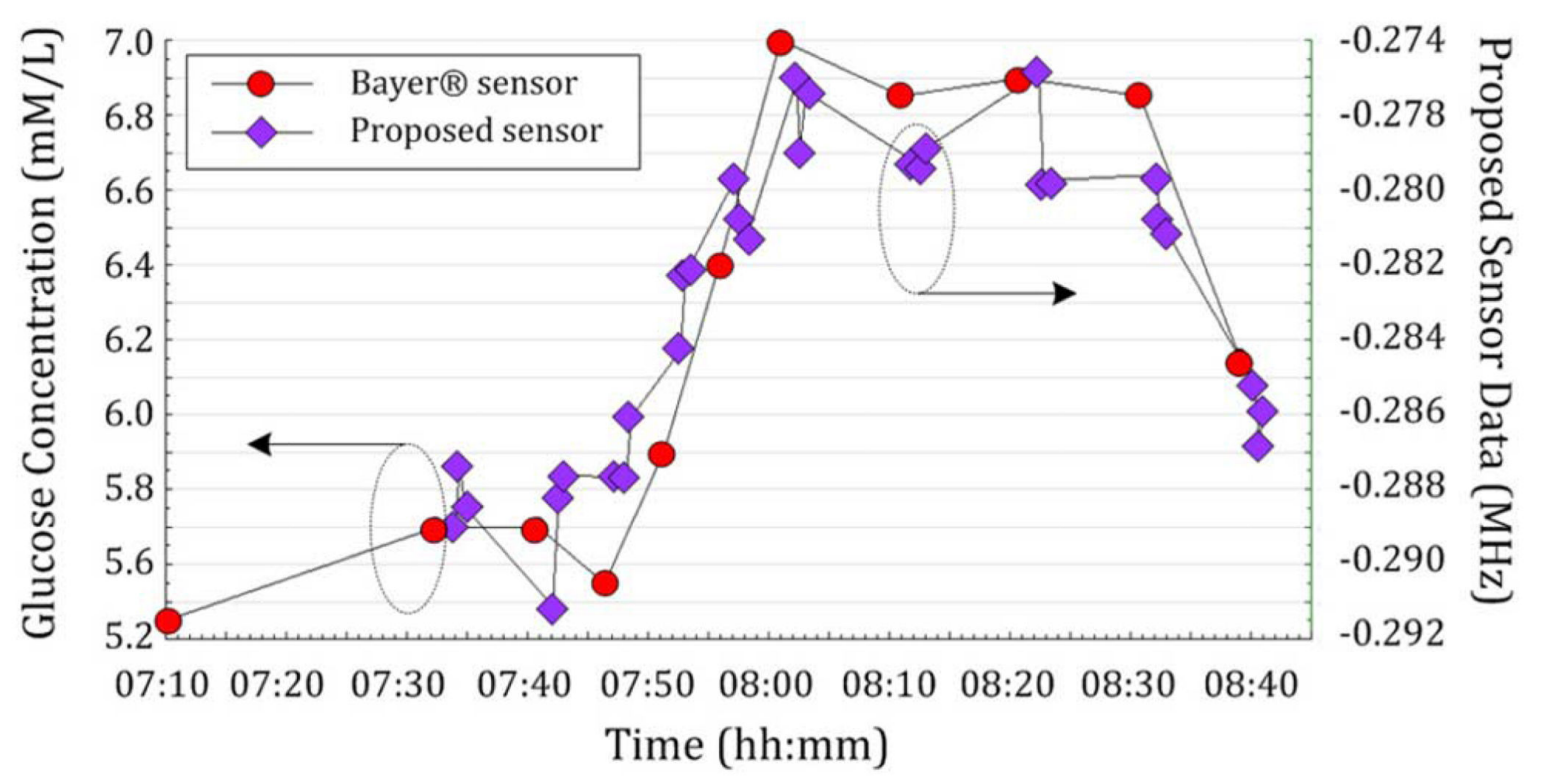
OGTT I. The sensor response (here the change in bandwidth) is compared with blood glucose levels measured using a “blood-strip” glucometer (Bayer CONTOUR).

**Fig. 8:**
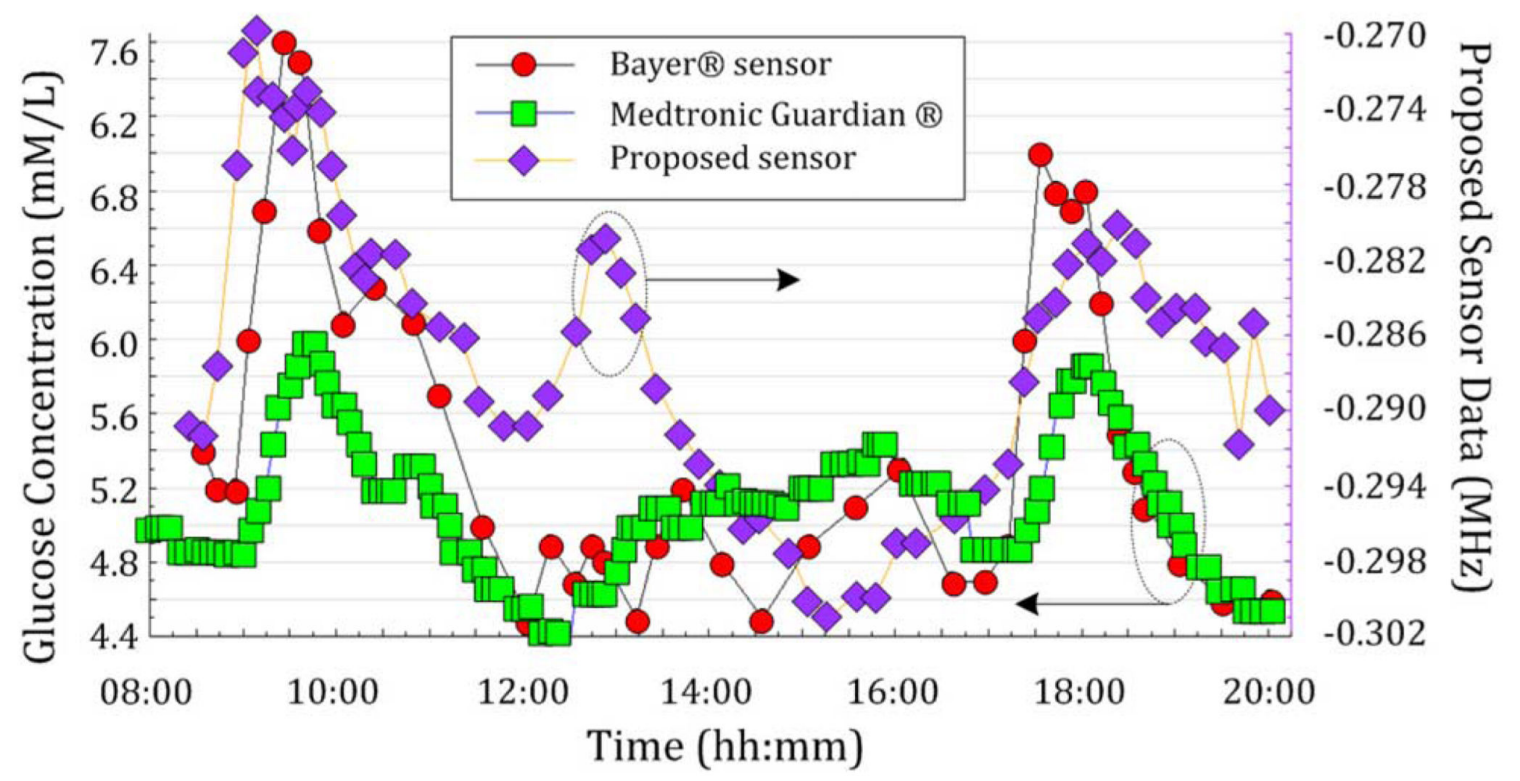
OGTT II. Sensor data (change in bandwidth) are measured over a continuous 12-h period involving three food ingestions, and are compared with data from a continuous glucose meter (Medtronic Guardian CGM) and blood-strip glucometer (Bayer CONTOUR), after [27].

Two different *in vivo* test results of the proposed sensor on a single nondiabetic subject are shown in Figs. 7 and 8. Here we measure the 3-dB bandwidth changes from our sensor that are temperature corrected using the reference ring. We observe a change in complex permittivity of the body as a result of glucose content in the tissue directly beneath the sensor. *In vivo* tests were carried out on a single nondiabetic individual. In Fig. 7, blood strip sensor readings are compared with the proposed microwave sensor data. Each point (diamond) represents an averaged 3-dB bandwidth from a burst of ten frequency sweep data. This is repeated three times per blood strip measurement. Although some variation is observed within the three measurements, the data show good precision in general. Such OGTT results are often presented in the context of NIBGM, but are not necessarily indicative of a successful device owing to other physiological changes that are triggered, e.g., in body temperature and blood salinity.

The measurements in Fig. 8 cover a period of 12 h with three separate food intakes (i.e., breakfast, lunch, dinner). Each point (diamond) represents an average of three 3-dB bandwidth values, each extracted from a burst of ten frequency sweep data. In this result, it is notable that the peak immediately after lunch is not clearly represented by the commercial continuous glucose monitor (square) or even by the blood strip measurement (circle), while microwave data (diamond) clearly show a peak at the expected time frame after lunch. Although most widely used, the accuracy of the glucose meter can be easily affected by several factors, including calibration, temperature, quality of blood sample, high levels of interferents, humidity, and aging of test strips [31].

This provides a far more precise picture than that obtained from an oral glucose tolerance test because results from different blood glucose episodes can be quantified next to each other. Many other techniques attempting to solve the same problem of noninvasive blood glucose monitoring examine only one food cycle. In our experience this is not sufficient to evaluate the effectiveness of the measurement method in question. Note that the subject is nondiabetic so the blood glucose levels here are in the very limited range between 4.5 to 7.6 mmol/L (i.e., 80–150 mg/dL).

## III. *In Vitro* Interference Test

### A. Measurement Setup

The Franz cell was first introduced in 1975 to systematically validate percutaneous absorption of human skin *in vitro* [32], and has become the most widely used method in understanding the permeation characteristics of synthetic or biological membrane in, for example, transdermal drug delivery [33]. Typically, a Franz cell consists of two separate chambers, a donor chamber on the top and receptor chamber on the bottom, with the membrane under test placed horizontally between the two chambers. The donor chamber is filled with the material to be permeated through a test membrane, and the sample is obtained from the sampling arm in the receptor chamber. For temperature control, the receptor chamber is surrounded by a temperature jacket with inflow and outflow arms, which is filled with water.

**Fig. 9:**
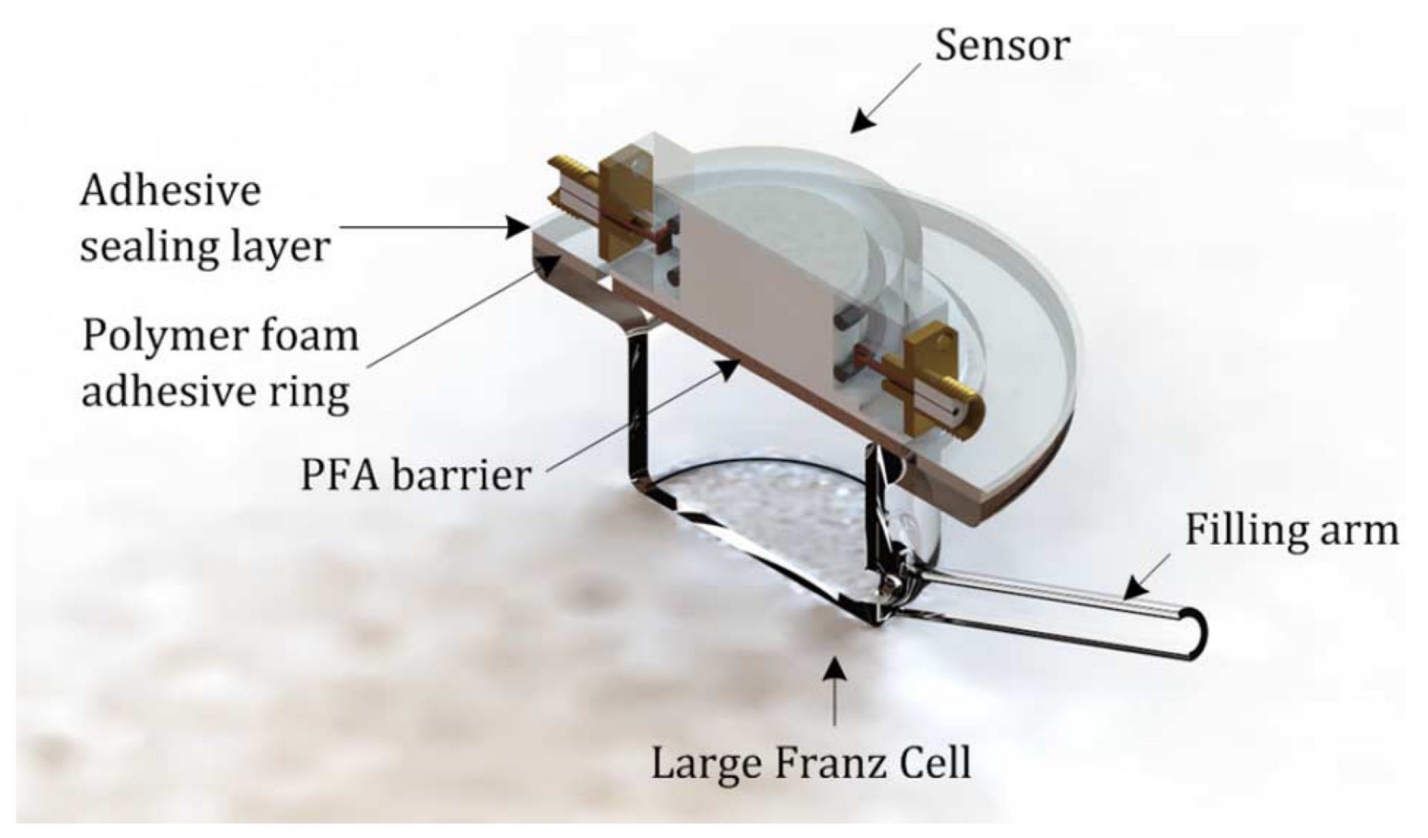
3-D cross-section view of the Franz Cell—sensor assembly.

In this experiment, since the goal is to monitor the change in dielectric properties due to glucose and other interferents injection by using the proposed sensor, some adjustment is made in the Franz cell assembly. The donor chamber is replaced by the proposed sensor, and the membrane under test is replaced by the Perfluoroalkoxy copolymer (PFA, Goodfellow) barrier. The sampling arm is used as a filling arm to inject glucose and other interferents. Franz cells with 40-mm receptor orifice diameter and a volume of approximately 21 mL were chosen for the *in vitro* experiments, as shown in Fig. 9. The use of the proposed double split-ring sensor is preferable as it makes these experiments more relevant and more readily interpreted by direct comparison to *in vivo* studies. Before the experiment measurement, small adjustments have to be made to the double split-ring sensor. The rings are moved further way from the sample to match better the conditions of the Franz cell since water has a higher microwave loss per unit volume than living tissue; hence, the *in vivo* arrangement would otherwise suffer from excessive absorption of the EM field.

**Fig. 10:**
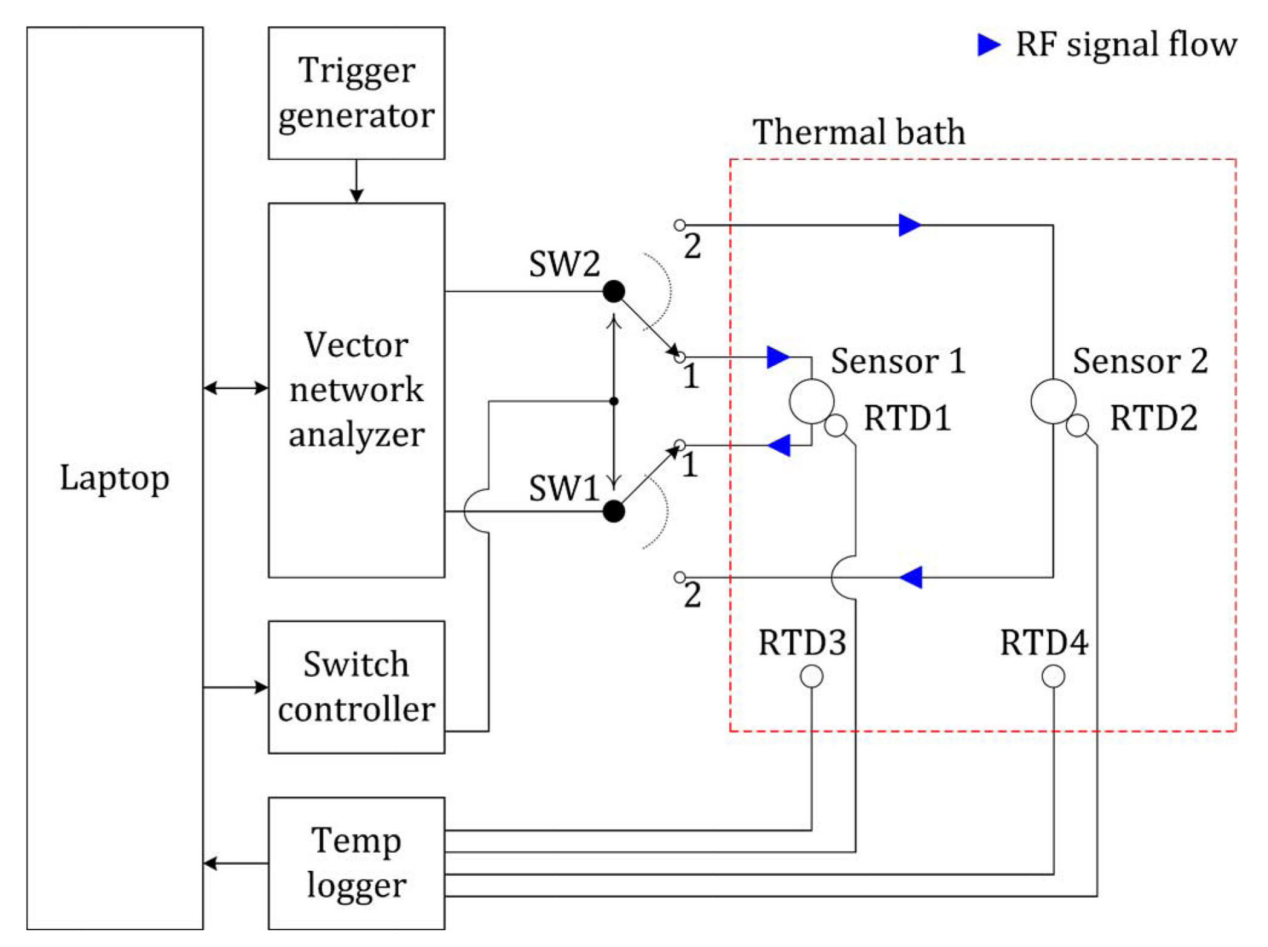
Schematic diagram of the measurement setup (resistance temperature detector: RTD).

Fig. 10 shows the schematic diagram of the measurement setup. A primary thermal bath contains the sensors and four RTDs. The temperature of the thermal bath and of the sensor housing is continuously monitored using platinum resistance thermometers in order to ensure stable operating conditions during the experiment. RTDs 3 and 4 are mounted on a raised stainless-steel perforated plate mounted in the bottom of the thermal bath. RTDs 1 and 2 are mounted directly on the sensor shield. The temperature at each RTD is logged continuously by the laptop during experiments. The Lab armour beads completely fill the bath and are poured over the sensors to completely submerge them before measurement, leaving only the Franz cell filling arm exposed. A secondary thermal bath (not shown) is used to keep all reagents required for injection into the cells ready at 37 °C.

The two sensors are multiplexed using two switches controlled by a digital output of a multifunction I/O unit that is interfaced to the laptop via USB. When the switches are in the first position, the VNA measures Sensor 1, and when in the second position, the VNA measures Sensor 2. The VNA is set to begin each measurement on an external trigger signal. This is provided by an external signal generator set to output square-waves with a frequency of 1 Hz. Thus, each sensor is measured alternately every 2 s.

**Fig. 11:**
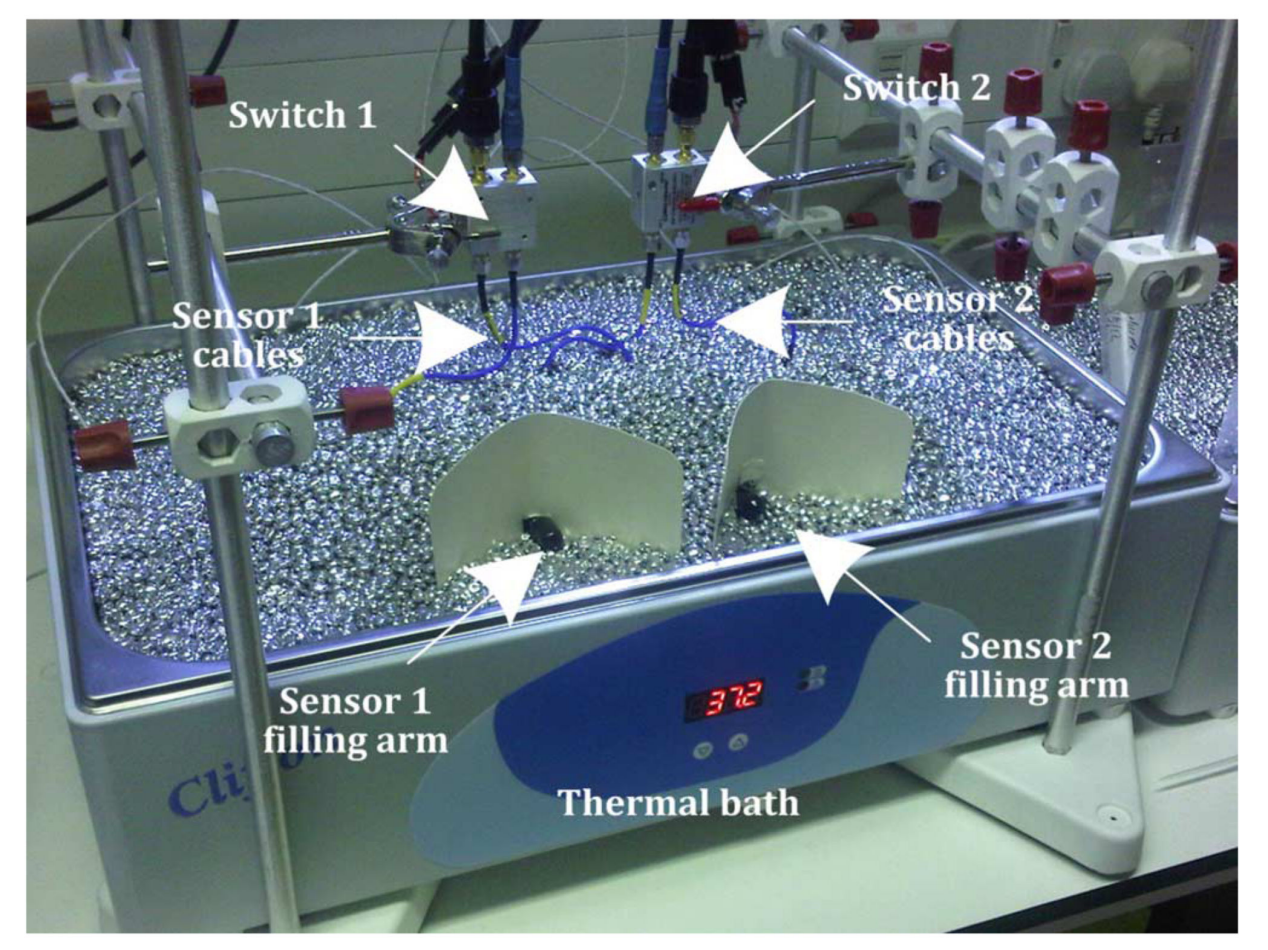
Photograph of the measurement setup. Two sensors are immersed in an aluminium bead thermal bath. The glucose and interferent solutions are injected to the exposed sampling arm of one Franz cell.

Two split-ring sensors are fitted to two Franz cells so that one could function as a reference sensor if required (with no added interferents). The bath is set to 37 °C providing a mean sensor temperature of 34 °C. Furthermore, no stirrer bar is used due to its interference with the microwave sensor, caused by the movement of its high permeability magnet material. A photograph of an individual sensor and Franz cell arrangement and the pair of sensors immersed in the thermal bath are shown in Fig. 11.

### B. Sample Preparation and Experiment

Initial Franz cell measurements are made using an inert membrane of 1-mm thickness. PFA (Goodfellow) is chosen, as it has a negligible effect on the chemistry of the cells or on the EM field of the sensor. Cells are initially filled with phosphate buffered saline (PBS). The PFA layer is sealed using vacuum grease. The ring sensors are secured to these cells using an adhesive membrane (CoTran 9720 backing, 3M). The loaded cells are left overnight to thermally equilibrate. Both sets of cells and sensor are measured simultaneously using an electronic microwave switch arrangement. One cell is maintained with PBS solution only to act as a baseline reference where necessary.

A 1-M stock solution of glucose is prepared by dissolving glucose powder (G7528, Sigma-Aldrich), in high-performance liquid chromatography (HPLC) grade water (Fisher). Glucose solution is then spiked into the Franz cells in 200-*μ*L aliquots, up to 600 *μ*L, following removal of the same volume of receptor phase solution. Each injection of concentrated glucose solution thus increases the glucose concentration in the Franz cell by approximately 28 mM, based on a cell volume of approximately 21 mL. After each injection, the experiment is left for 30 min to equilibrate in temperature and concentration.

Due to the prolonged setup time of the cells, it was decided to combine interferent experiments. Thus, on the assumption that there is no interaction between interferents (a fair assumption given that they are normally coexistent in blood), the interferent and glucose experiments are divided into two separate experiment series that can be run without disassembly. Stock solutions of interferent are produced by dissolving powders (99%, Sigma-Aldrich) in HPLC grade water. The concentrations of the various interferents are compounded as the sequence progressed and the relative changes in sensor readings are taken. The series shown in Table III are then used as the basis for all subsequent Franz cell studies.

Due to the high limit of detection of glucose with this particular experimental setup, caused by a number of factors discussed in Section III-C, the concentrations used for these experiments are chosen to be much higher than the nominal ranges found in blood. All microwave sensors of our type will not be specific to glucose, so in order to generate meaningful data, the concentrations of all interferents are extended up to three times those initially found in the human body. Using a minimum of three concentration values allows for any correlation between sensor response and interferent to be more readily discerned. Glucose concentration is also extended far beyond the clinical range so that its effect could be reliably detected before each experiment. Thus, interferent experiment series 2 is conducted with a constant glucose concentration of 84 mM. To optimize resources it is decided to focus on endogenous interferents only.

### C. Results and Discussion

**Fig. 12:**
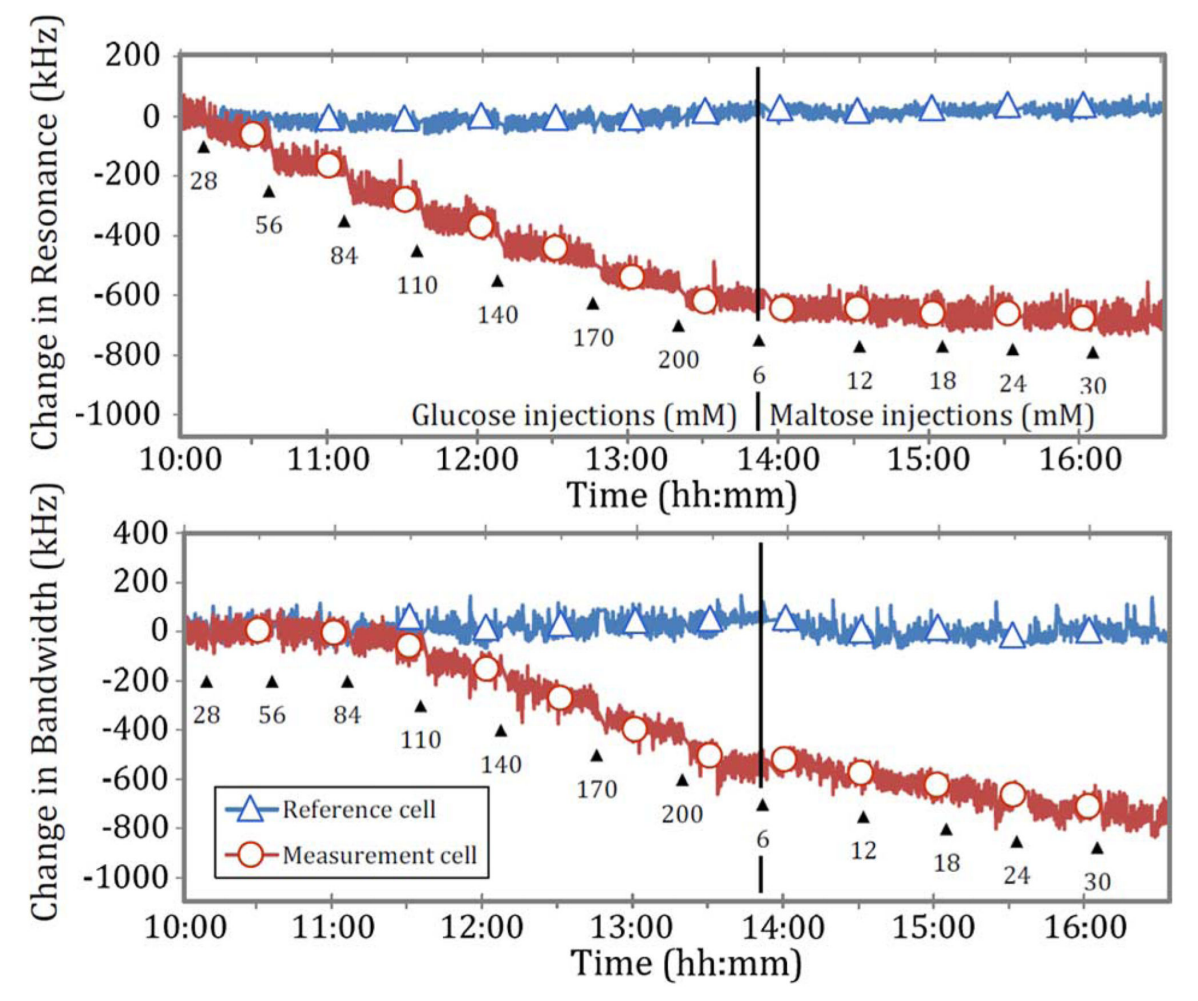
Change in resonance frequency and change in bandwidth over time with glucose and maltose injection, according to experiment series 1 in Table III.

Data for experiment series 1 in Table III are shown in Fig. 12. The level of noise is two orders of magnitude higher than that experienced when the Franz cell is empty, indicating that its origin is due to the presence of the solution in the cell. This noise occurs because the solutions’ main constituent, water, has a dramatic effect on the microwave EM field, which makes this experimental setup sensitive to several perturbing factors.

Primary among these is temperature, which causes the permittivity of water to decrease by 1% per °C increase in temperature. Although the temperature of the aluminium shield is continually monitored and typically shows fluctuations of less than 0.1° over a 30-min period, temperature sensitivity to the liquid solution is so high that such fluctuations can explain the large amplitude of the noise seen. This explanation is further reinforced by the distribution of the noise spectral energy toward low frequencies, as would be expected for a thermal system with a large time constant. The low sensitivity to glucose found in these inert barrier reference experiments compared to that found *in vivo* (around 3 kHz/mM here, as opposed to 100 kHz/mM *in vivo*) means that this level of noise becomes significant. Temperature control with this precision is not an issue *in vivo* due to the unexpectedly high responsivity to glucose found in previous *in vivo* experiments.

It would be difficult to maintain greater temperature stability than this in these experiments. Furthermore, due to the confines of the Franz cell geometry, it is not practical to measure the temperature of the solution directly. Even if this were possible, few temperature measurement devices have a sensitivity as acute and response time as immediate as the microwave resonant sensor itself. Thus, temperature correction via thermometry is unhelpful. Another possible solution to temperature correction is to use the secondary mode of resonance of the double split-ring resonator as a reference. However, because both resonant sensor modes are in a regime where fluctuations are dominated by the sample itself, any fluctuations due to temperature are inseparable from those due to concentration of measurand solute, making temperature correction using reference modes also unhelpful. Thus, after much effort to reduce the level of fluctuations to a minimum level, it is decided to proceed and to increase the concentration of glucose to a resolvable level at three times that originally planned, and similarly scale the interferent concentrations in accordance.

**Fig. 13:**
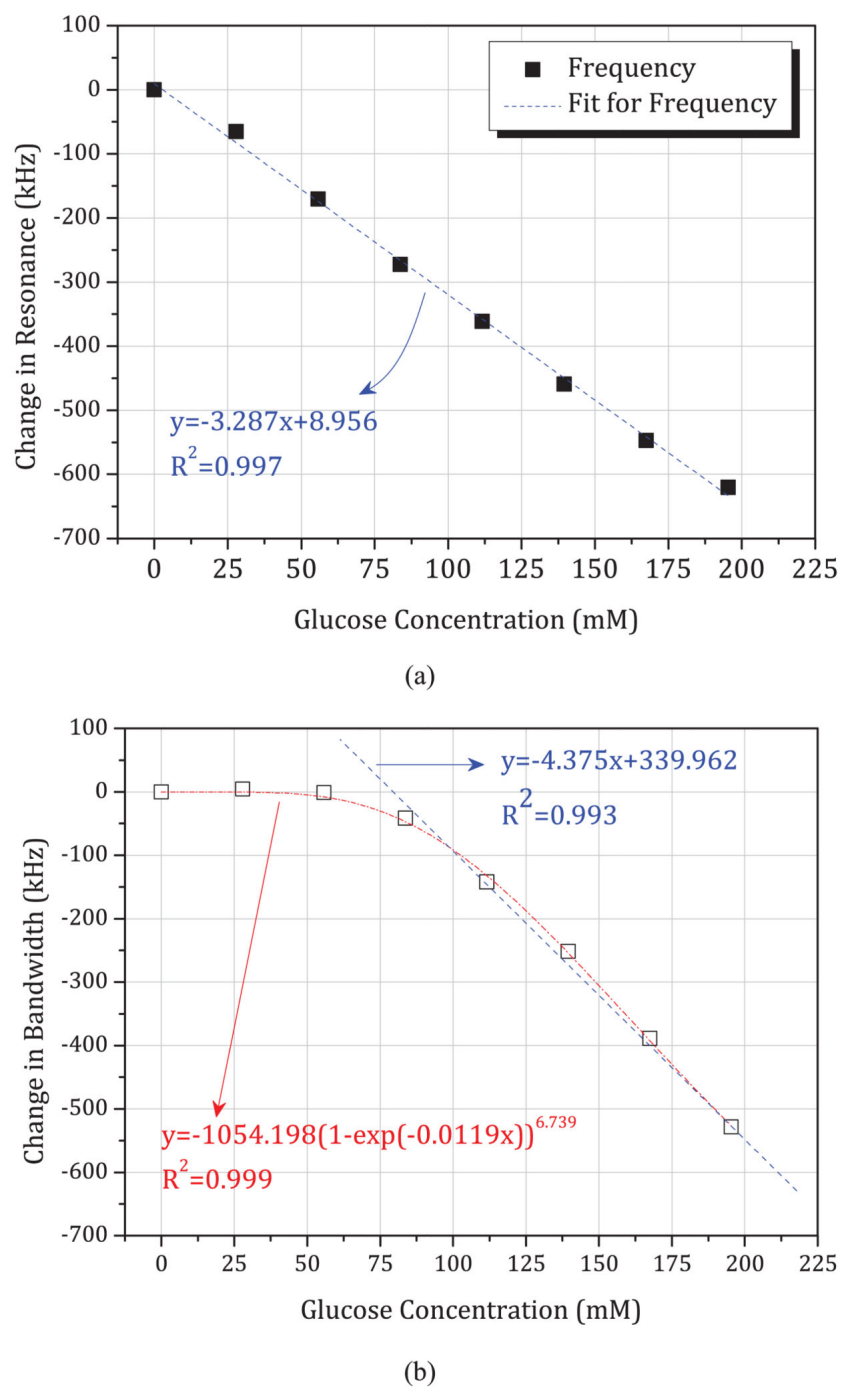
Linear correlation showing sensitivity between: (a) resonance frequency change and glucose concentration and (b) bandwidth change and glucose concentration, from experiment series 1.

**Fig. 14:**
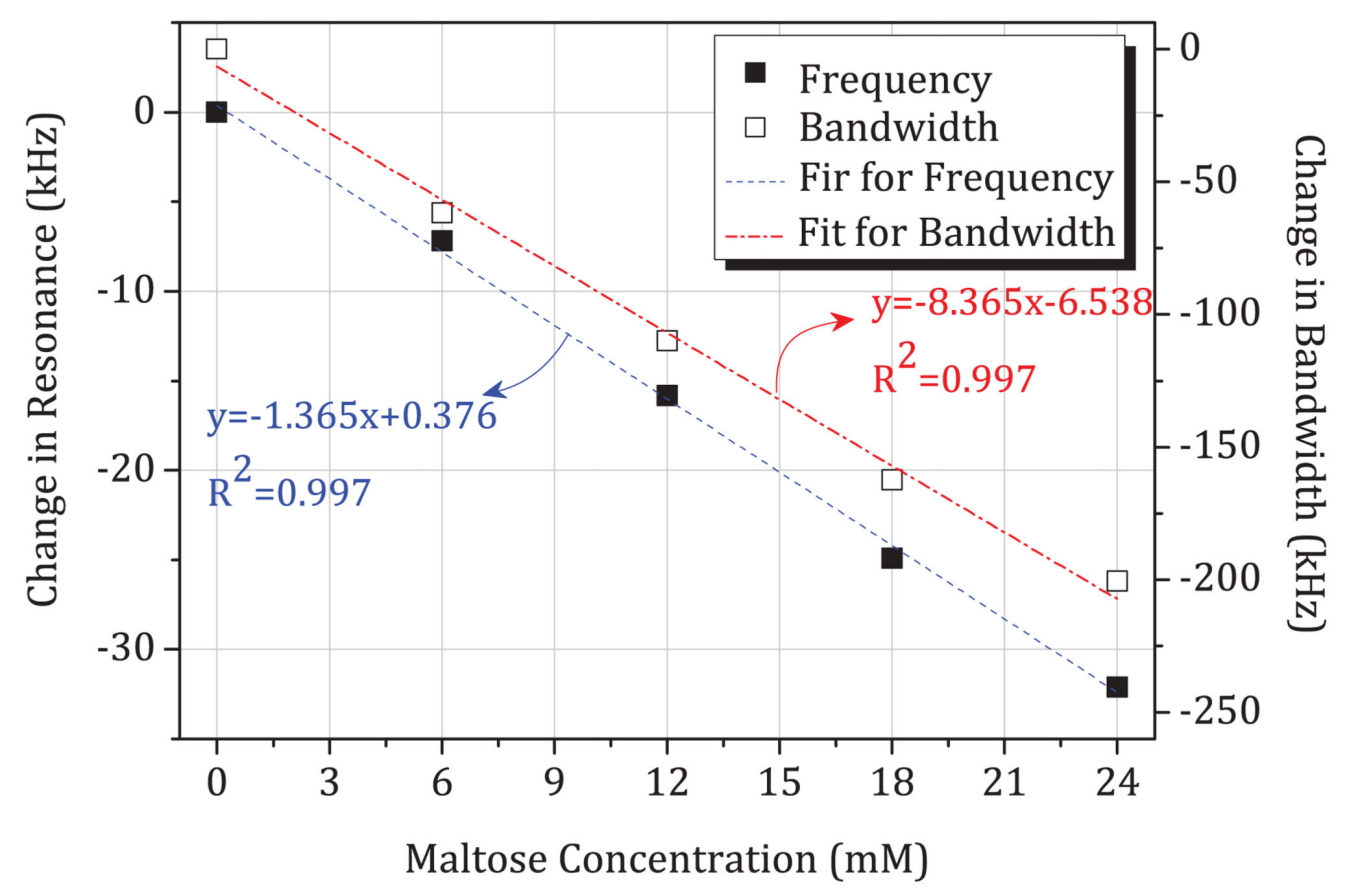
Sensitivity of resonance frequency change and bandwidth change over maltose concentration, from experiment series 1.

The time-domain data of Fig. 12 are re-plotted in Figs. 13 and 14, where we plot directly the change in resonant frequency and change in bandwidth as a function of glucose and maltose concentration, respectively. In Fig. 13(a), we note that these changes are small, approximately −3.287 kHz per mM in resonant frequency. This is smaller by an order of magnitude and in the opposite direction compared to those measured previously during *in vivo* studies of blood glucose using the same sensors (approximately + 100 kHz per mM in resonant frequency). We observe a linear decrease of resonant frequency with increasing glucose concentration, as shown in Fig. 13(a). The sensor can thus measure glucose linearly based on resonant frequency. However, the noise level is such that the limit of detection is at least 30 mM when sampling once every second (based on three times the root mean square noise amplitude of 30 kHz). The resonant bandwidth, although it shows linear correlation after 80 mM, is a nonlinear function of glucose concentration and shows very little variation over clinically important concentrations, up to 30 mM. Slope of change due to maltose is even smaller than due to glucose, showing −1.365 kHz per mM in resonant frequency, as shown in Fig. 14. However, the *in vitro* slope of change in bandwidth is −8.365 kHz per mM, which is larger than per mM in glucose concentration.

**Fig. 15:**
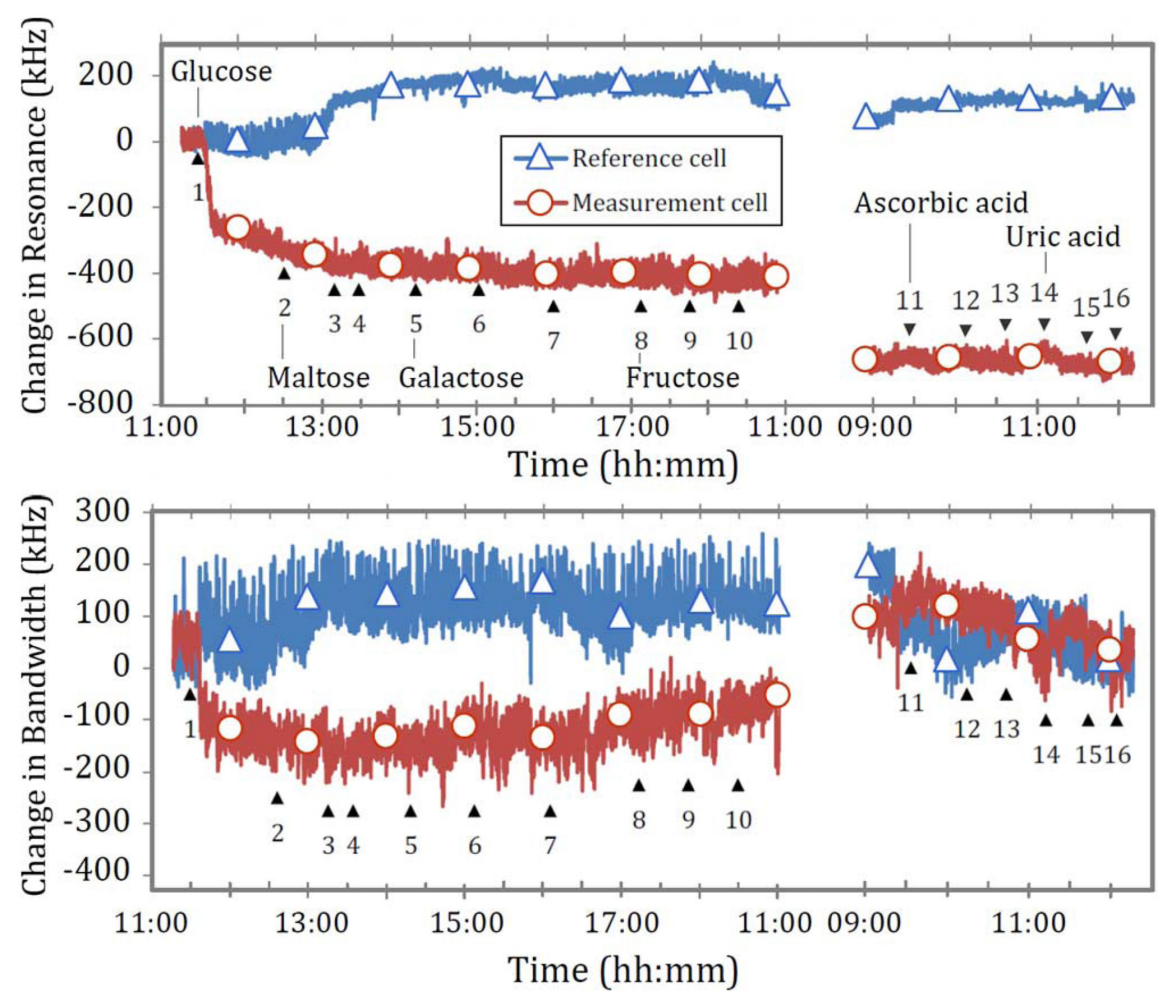
Change in resonance frequency and bandwidth over time with glucose and interferents, according to experiment series 2 in Table III.

Fig. 15 shows the interferents from experiment series 2 in Table III measured over time. Data are split over the course of two days to better observe any long-term effect of earlier injection (injection 1–10 in experiment series 2). In the change in resonance trace, the initial glucose injection is apparent as a large initial step in the measurement cell sensor readings. All subsequent interferents appear to have negligible effect on sensor readings at the concentrations measured. Thus, one can draw a provisional conclusion that none of the interferents tested has any direct and significant effect on glucose measurement, even at concentrations several times that found in blood.

## IV. Conclusion

In this paper, a noninvasive and continuous blood glucose monitoring sensor topology based on microwave technology has been proposed and validated with *in vivo* and *in vitro* interference tests. The results compare favorably with commercial invasive blood glucose monitors. It should be stressed that the test subjects in this work are nondiabetic so only a narrow range of blood glucose levels are involved here. We next plan to conduct clinical trials on people with diabetes, which will extend the range of glucose values to populate fully the consensus error grid and test more fully the application of our sensor to this hugely important healthcare problem.

By careful measurements of a sensor adapted to fit a Franz cell, we have investigated the effects of increasing glucose concentration on the sensor response and studied the effects of endogenous interferents common to all subjects, i.e., common sugars, vitamins (ascorbic acid), and metabolites (uric acid). With an inverted membrane, we measured the subtle changes in the water response caused by dissolved species, which give extremely small sensor responses, even for concentrations well above those that are clinically relevant. Now we have shown that maltose, fructose, and galactose also produce only small changes, at least three times less than the effects of glucose, and that other endogenous interferents have no significant direct effect. Continued interference studies, including further exogenous interferers (e.g., pharmaceuticals) will be performed as part of ongoing work if necessary and of value.

The fact that *in vitro* glucose measurements produce measurable signals at unreasonably high glucose readings (more than 50 mM/L) compared with the *in vivo* studies is a curiosity of these microwave measurements that is consistent the observations of others. We conclude that the *in vivo* measurement of glucose using this microwave technique is not a direct one, and this merits further investigation as to the mechanism of the glucose modification of the dielectric property of human tissue.

## Figures and Tables

**TABLE I:** Dielectric Properties of Each Layer

	Conductivity (S/m)	Relative permittivity	Loss tangent
Fat	0.065	5.395	0.154
Muscle	1.142	54.112	0.271
Skin	1.036	39.661	0.335

Measured data at 1.4 GHz at 37°C

**TABLE II:** EM Simulation and Measurement Results, When the Sensor is on the Body

	EM simulation	Measurement	Unit
f_0_	1.433	1.431	GHz
3dB BW	16.6	19.1	MHz
Peak S_21_	−39	−41	dB
Q	86	75	

**TABLE III:** Type of Interferents and List of Injection

	Experiment series 1			Experiment series 2		
Injection	Stock solution	Resultant conc.	(mM)	Stock solution	Resultant conc.	(mM)
1	Glucose	28	±2 %	Glucose	84	±2 %
				
2	Glucose	56		Maltose	6	
3	Glucose	84		Maltose	12	
4	Glucose	111		Maltose	18	
				
5	Glucose	140		Galactose	2	
6	Glucose	167		Galactose	4	
7	Glucose	195		Galactose	6	
		
8	Maltose	6		Fructose	1	
9	Maltose	12		Fructose	2	
10	Maltose	18		Fructose	3	
				
11	Maltose	24		Ascorbic acid	0.2	
12	Maltose	30		Ascorbic acid	0.4	
			
13				Ascorbic acid	0.6	
				
14				Uric acid	0.2	
15				Uric acid	0.4	
16				Uric acid	0.6	
